# Impact of anti-HER2 therapies on potentially avoidable deaths from stage II and III HER2-positive breast cancer in the Brazilian public healthcare system

**DOI:** 10.1016/j.breast.2026.104940

**Published:** 2026-10-01

**Authors:** André Mattar, Francisco Pimentel Cavalcante, Nelson Francisco Correa-Netto, Iago Silveira, Eduardo Baldassin Pavanel, Cintia S. Kurokawa La Scala, Patricia Klarmann Ziegelmann

**Affiliations:** a Hospital da Mulher, São Paulo, SP, Brazil; b Hospital Geral de Fortaleza, Fortaleza, CE, Brazil; c Produtos Roche Químicos e Farmacêuticos, São Paulo, SP, Brazil; d Universidade Federal Do Rio Grande Do Sul (UFRGS), Porto Alegre, RS, Brazil

**Keywords:** HER2-Positive breast cancer, Access to care, Treatment inequity, Health technology assessment

## Abstract

**Purpose:**

Limited access to contemporary anti-HER2 therapies in Brazil's public healthcare system (Sistema Único de Saúde -SUS) may contribute to avoidable mortality among women with HER2-positive early breast cancer. We estimated the number of potentially preventable deaths associated with incorporating adjuvant trastuzumab emtansine (T-DM1) and perioperative pertuzumab into SUS.

**Methods:**

We developed a static decision-tree microsimulation model with an 8-year time horizon. The study population was derived from nationwide real-world data from DATASUS, which captures all patients treated within SUS. Three treatment strategies were evaluated: (1) current SUS standard (perioperative trastuzumab only), (2) incorporation of adjuvant T-DM1 for patients with residual invasive disease after neoadjuvant therapy, and (3) a gold-standard regimen including perioperative pertuzumab and response-guided adjuvant T-DM1. Clinical inputs were obtained from the NeoSphere, APHINITY, and KATHERINE trials. Probabilistic sensitivity analysis with 100,000 simulations was performed to estimate mortality outcomes and 95% confidence intervals (CIs).

**Results:**

A total of 22,432 women with stage II–III HER2-positive breast cancer were identified. The projected mean number of deaths at 8 years was 3606 (95% CI, 3082–4180) under the SUS standard, 2672 (95% CI, 2244–3147) with adjuvant T-DM1, and 2390 (95% CI, 2034–2790) with the gold-standard regimen. Incorporation of T-DM1 was associated with 934 fewer deaths (25.9% relative reduction), whereas full adoption of the gold-standard strategy was associated with 1216 fewer deaths (33.7% relative reduction).

**Conclusion:**

Expanding access to adjuvant T-DM1 and perioperative pertuzumab within SUS could meaningfully reduce mortality and narrow disparities in outcomes for Brazilian women with HER2-positive early breast cancer.

## Highlights

• Analyzed 22,432 HER2+ breast cancer cases within the Brazilian public health system.
• We used a simulation model to compare three breast cancer treatment regimens.
• A targeted therapy could prevent 934 breast cancer deaths over eight years.
• Combining specific treatments may prevent 1216 deaths, a 34% reduction.
• Improved access to care lessens avoidable deaths and health disparities.

## Graphical abstract

**Figure:**
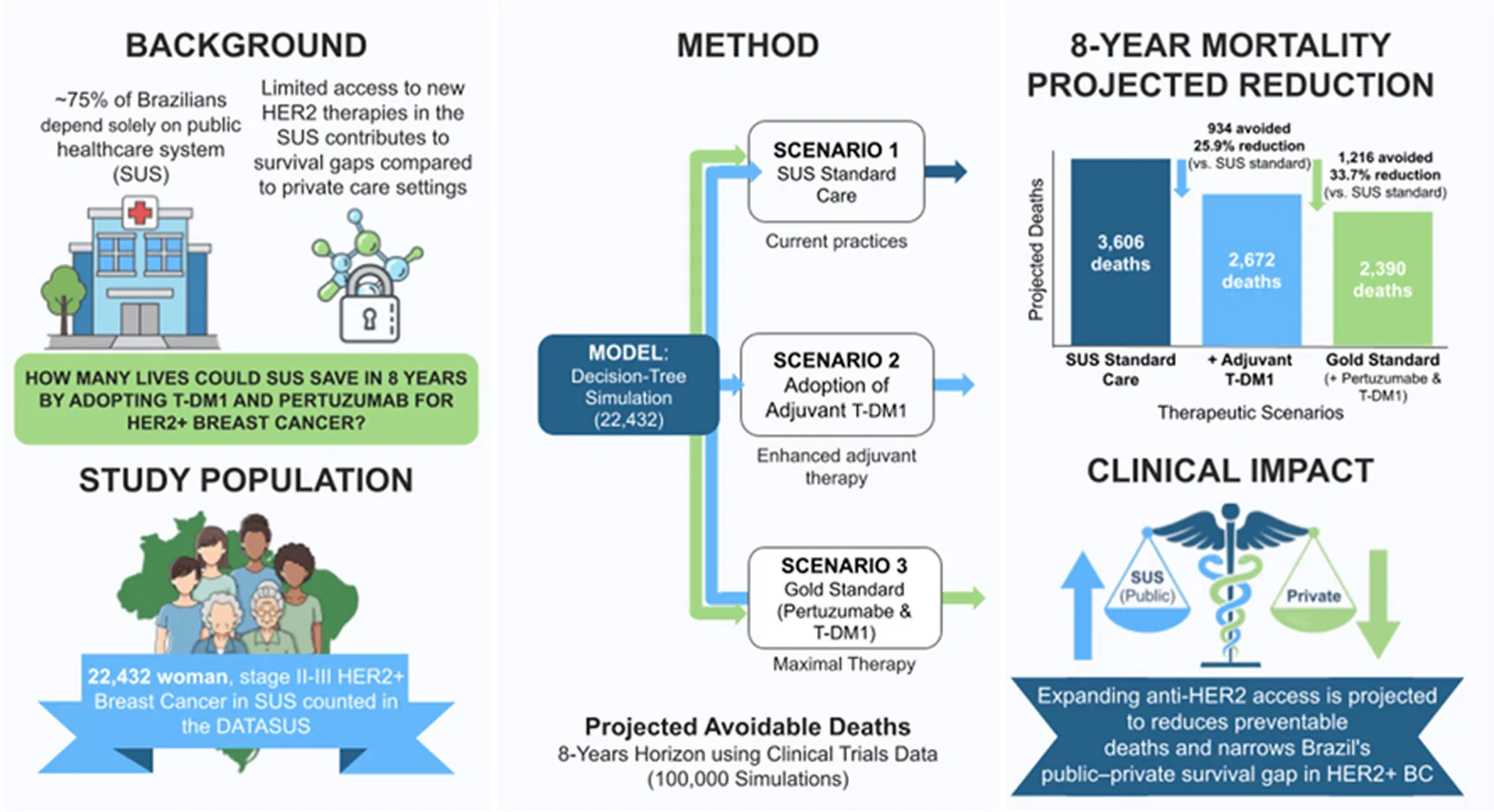

## 1 Introduction

In women, breast cancer is the most common cancer and the leading cause of cancer-related mortality worldwide, with an estimated 666,103 deaths globally in 2022^1^ [1]. In Brazil, 20,165 deaths from breast cancer were reported in 2023, mainly in the Southeast and Northeast regions [2]. Amplification or overexpression of human epidermal growth factor receptor 2 (HER2) occurs in approximately 15-30% of all breast cancers, a prevalence also observed in Brazil [3]. Historically, this subtype has been clinically aggressive, even in early settings. A Brazilian cohort demonstrated a lower 5-year overall survival (OS) for stage II (82·34%) and III (64·10%) HER2-positive (non-luminal) disease, in comparison with those with luminal-like disease (97·59% and 85·83%, respectively) [4]. However, the prognosis of the HER2-positive subtype has been significantly improved after the introduction of HER2-targeted therapies, both in the metastatic and early-stage settings [5].

The benefit of adding the HER2-targeted monoclonal antibody trastuzumab for patients with HER2-positive early breast cancer was established in clinical trials of adjuvant therapy, with a significant increase in disease-free survival (DFS) and OS [[6], [7], [8]]. The further addition of pertuzumab in the neoadjuvant setting improved the pathological complete response (pCR) rate [9,10]. Furthermore, the Phase III APHINITY trial demonstrated that combining pertuzumab to adjuvant trastuzumab and chemotherapy increased OS and invasive disease-free survival (IDFS), particularly in patients with node-positive disease (hazard ratio [HR] = 0·79; 95% confidence interval [CI]: 0·64–0·97 for OS; and HR = 0·74; 95% CI: 0·62–0·88 for DFS) [11]. More recently, trastuzumab emtansine (T-DM1), an antibody-drug conjugate combining trastuzumab with the cytotoxic agent emtansine, yielded a significant 34% decrease in the risk of death versus trastuzumab for patients with residual invasive disease following neoadjuvant therapy in the KATHERINE trial (HR = 0·66; 95% CI: 0·51–0·87; P = 0·003) [12].

Despite this progress in systemic treatment, many patients face substantial challenges in accessing optimal therapy. This is particularly relevant to the Brazilian dual healthcare system, which is composed of public and private sectors [13]. The Unified Health System (Sistema Único de Saúde, SUS) provides universal, publicly funded coverage and is responsible for delivering care to the vast majority of the population [13]. Approximately 75% of Brazilians (more than 160 million people) depend exclusively on SUS for cancer diagnosis and treatment [14]. However, the coexistence of public and private care generates substantial inequities in access to high-cost oncologic therapies, with faster adoption and broader availability in the private sector [14,15]. Despite its universal mandate, SUS faces persistent resource restrictions and operational challenges that often delay the incorporation of innovative treatments and limit timely access to state-of-the-art care [14,15]. For a drug to be incorporated and available in SUS, after a Brazilian Health Regulatory Agency (ANVISA) approval, it must undergo a rigorous evaluation by the National Committee for Health Technology Incorporation (CONITEC), which includes analysis of regulatory compliance, efficacy, safety, economic evaluation, and budget impact [16]. This process results in a significant disparity in the availability of novel anti-HER2 therapies between the public and private healthcare sectors in Brazil. For example, despite CONITEC's recommendation to incorporate adjuvant T-DM1 in September 2022, the drug only became available for SUS patients in November 2025, marked by its delivery to the Brazilian Ministry of Health (MoH), representing a delay of more than 3 years since the initial recommendation [17]. Moreover, perioperative pertuzumab is not publicly funded and remains unavailable in SUS to this day. In contrast, the private sector already has regulatory approval and broad access to both drugs. The restricted access to optimal therapies is not exclusive to Brazil but also observed in other low- and middle-income countries with resource constraints. Given the significant clinical benefit of these therapies, particularly on OS, this limited access may negatively impact patient outcomes, especially their survival [18,19].

The concept of avoidable deaths has been widely used to assess the impact of disparities in access to different health interventions and policies, quantifying mortality that could be averted under a more favorable scenario. This metric is typically estimated as the difference in the number of deaths between two compared scenarios. The OECD and Eurostat, for instance, apply this concept assuming that premature deaths occurring below a given age (<75 years) are avoidable through effective primary prevention and timely healthcare interventions [20]. Rutherford et al. estimated avoidable deaths as the difference in mortality that would result from eliminating socioeconomic inequalities in breast cancer survival in England and Wales [21], while Langselius et al. more recently applied this approach on a global scale, estimating the deaths that could be averted among patients diagnosed with cancer through both primary prevention and improved early detection and access to curative treatment [22].

Therefore, this study aimed to estimate the number of avoidable deaths that could be averted through the potential incorporation of novel HER2-targeted therapies for women diagnosed with stage II and III HER2-positive breast cancer treated in the Brazilian public healthcare system (SUS).

## 2 Methods

### 2.1 Model description

A static decision tree model with an 8-year time horizon was developed using simulation to estimate the number of potentially avoidable deaths among Brazilian women diagnosed with stage II and III HER2-positive breast cancer under three alternative treatment scenarios (Fig. 1). These avoidable deaths were calculated as the difference in the cumulative number of model-projected all-cause deaths within the 8-year time horizon between the same cohort treated under two alternative treatment scenarios. These estimates represent deaths potentially averted through improved access to curative systemic anti-HER2 therapy under the assumptions of the model, rather than deaths directly observed or individually classified as avoidable. Notably, this analysis does not account for deaths preventable through primary prevention, screening, earlier diagnosis, or improvements in other cancer care components.

**Fig. 1:**
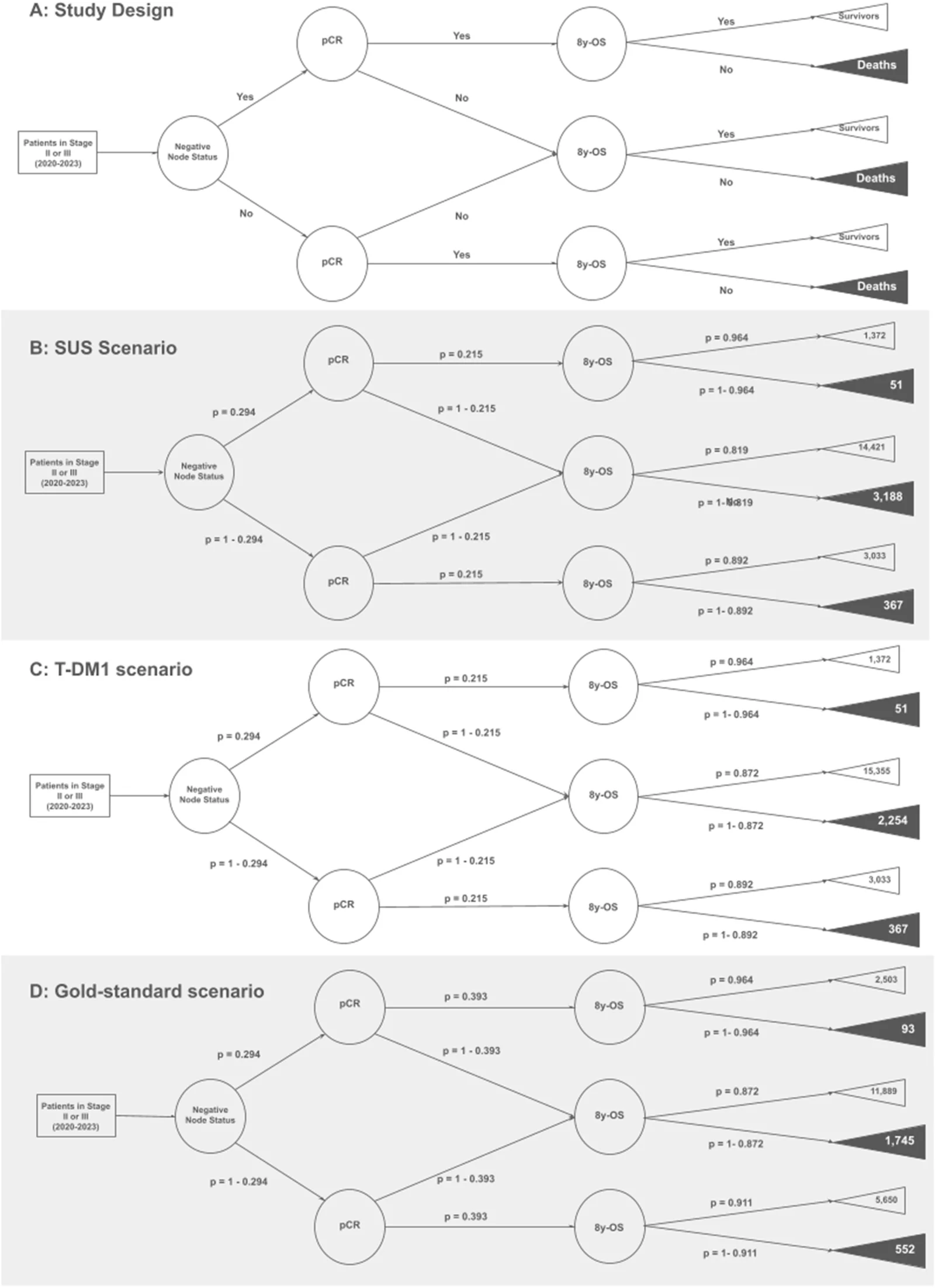
Decision-tree model comparing treatment scenarios for patients with stage II–III breast cancer in the Brazilian Unified Health System (SUS) and the number of avoidable deaths.

### 2.2 Study population and data source

The study population was identified through a cross-sectional analysis using data from the Department of Information and Informatics of the SUS (DATASUS), a national registry that includes all patients treated in the public health system. The analysis used records of outpatient chemotherapy for patients with breast cancer (International Classification of Diseases-10 [ICD-10] C50). These records were extracted from high-cost chemotherapy procedure authorizations (APAC-AQ) files of Ambulatory Information System (SIA-SUS). In DATASUS, HER2-positive non-metastatic breast cancer treated with neoadjuvant systemic therapy can be identified with high specificity through the APAC-AQ code, which is uniquely assigned to HER2-positive, stage II or III, non-metastatic patients receiving neoadjuvant trastuzumab-based therapy within SUS. This procedure code is not used for metastatic disease, private-sector care, or other breast cancer subtypes, providing a validated operational proxy for identifying the population of interest within the administrative database. Patient records were provided on an individualized and anonymized basis. Eligible patients were women aged 18-99 years at diagnosis, with a first treatment for stage II or III HER2-positive breast cancer (ICD-10C50) registered in the DATASUS database between January 2020 and December 2023 (see “APAC-AQ codes for study eligibility” in Supplementary Materials). This period was chosen due to ANVISA approval of adjuvant T-DM1 in 2019 in Brazil, and the availability of the most recent fully recorded data at the time of analysis (June 2025).

No a priori sample size calculation was performed. Instead, a population-based approach was adopted, in which all eligible records identified in DATASUS during the study period were included, with no sampling or exclusions beyond the eligibility criteria described above. Because the cohort size is a fixed, fully enumerated input rather than a random sample, parameter uncertainty derives exclusively from the clinical data obtained from the pivotal trials, which was propagated through probabilistic sensitivity analysis.

### 2.3 Treatment scenarios

Three treatment scenarios were considered. 1) SUS scenario as the base case (standard treatment available in SUS until November 2025): neoadjuvant therapy including trastuzumab; adjuvant therapy with trastuzumab administered to all patients, irrespective of pathological response and lymph-node status. 2) T-DM1 scenario: neoadjuvant therapy including trastuzumab; adjuvant treatment with trastuzumab for patients with pCR, and with T-DM1 for those with residual invasive disease (non-pCR). 3) Gold-standard scenario (current standard-of-care): neoadjuvant therapy including trastuzumab and pertuzumab; adjuvant treatment as follows: trastuzumab for patients with pCR and no initial lymph-node involvement; trastuzumab and pertuzumab for those with pCR and positive lymph nodes before neoadjuvant therapy; and T-DM1 for those with residual invasive disease (non-pCR). To simplify the modelling structure, we refer to the combined use of pertuzumab in both the neoadjuvant and adjuvant settings as ‘perioperative pertuzumab.’ This terminology is used solely for consistency within the analytic framework and does not correspond to the terminology of the APHINITY trial, which evaluated pertuzumab exclusively in the adjuvant setting. In our model, perioperative pertuzumab therefore represents a guideline-concordant strategy in which patients receive pertuzumab alongside neoadjuvant trastuzumab plus chemotherapy and continue pertuzumab with trastuzumab as adjuvant therapy for patients with initial lymph-node involvement who achieve pCR.

### 2.4 Model parameters

The proportions of lymph-node involvement at diagnosis and pCR rates used in the model were based on results from the NeoSphere trial [9]. Eight-year OS probabilities after adjuvant treatment were obtained from trials corresponding to two settings of interest after neoadjuvant therapy. For patients with invasive residual disease, probabilities came from the KATHERINE trial, in which they were randomly assigned to adjuvant therapy with trastuzumab or T-DM1 [12]. For those with pCR after neoadjuvant therapy, probabilities came from the APHINITY trial, in which patients were randomized to receive adjuvant trastuzumab combined with pertuzumab or placebo [23].

The 8-year time horizon was selected to match the follow-up of the pivotal trials, which were designed to ensure an adequate cumulative number of overall survival (OS) events. Owing to the event-driven designs of these trials and the long natural history of early HER2-positive disease, earlier OS estimates were based on a low information fraction, yielding wide confidence intervals and limited statistical power to detect true differences; consequently, non-significant findings at shorter intervals should not be interpreted as an absence of benefit. All parameter values, distributions, and sources are reported in Supplementary Material S2 (Table S1).

### 2.5 Statistical analysis

Parameters’ uncertainty was incorporated through a probabilistic sensitivity analysis (PSA). All parameters - the probability of lymph-node involvement at diagnosis, the pCR rate in each scenario, and the 8-year OS probability applied to each branch - were varied jointly over 100,000 Monte Carlo simulations. Beta distributions were applied for all probability and OS parameters, with hyperparameters defined according to Briggs et al. [24].

In each iteration, the eligible cohort was distributed across the three decision tree branches - node-negative/pCR, node-positive/pCR, and non-pCR – based on the sampled probabilities of nodal involvement and pathological response for that scenario. Because each branch corresponds to a specific adjuvant treatment within a given scenario, the corresponding 8-year OS probability was applied to the allocated patients, yielding the expected number of deaths per branch; which were then summed to obtain the total number of deaths per scenario. Potentially avoidable deaths were calculated as the difference in total projected deaths between two scenarios, and the proportional reduction was defined as this difference divided by the number of deaths in the reference scenario. The full model notation, equations, and simulation algorithm are provided in Supplementary Material S3.

Three comparisons were evaluated: T-DM1 versus SUS, gold standard versus SUS, and gold standard versus T-DM1. Point estimates are reported as the mean across all iterations, alongside 95% credible intervals defined by the 2.5th and 97.5th percentiles of the simulated distributions. All analyses were performed in R, version 4.4.1.

### Ethical approval

This study was based exclusively on secondary analysis of publicly available, de-identified data from the Department of Information and Informatics of the Brazilian Unified Health System (DATASUS). The database contains anonymized administrative health records and does not include any identifiable personal information. According to Brazilian regulations (Resolution No. 510/2016 of the National Health Council), research using publicly accessible, de-identified data does not require review or approval by a Research Ethics Committee. Therefore, formal ethical approval and informed consent were waived.

## 3 Results

### 3.1 Baseline characteristics

A total of 22,432 patients with stage II and III HER2-positive breast cancer were identified in SUS and then considered in the estimation of avoidable deaths (Fig. 1 of Supplementary Material). The baseline characteristics of eligible patients are summarized in Table 1. At treatment start, most patients were 40-59 years old (57·0%) or 60-79 years old (27·3%). Within this cohort, the majority presented with stage III breast cancer (76·2%).

**Table 1:** Baseline characteristics of eligible patients.

	Overall N = 22,432 Patient, n (%)
*Disease stage*	
II	5341 (23·8)
III	17,091 (76·2)
*Age group*	
18-39 years	3148 (14·0)
40-59 years	12,777 (57·0)
60-79 years	6131 (27·3)
80-99 years	376 (1·7)

Table 2 presents the estimated number of patients and deaths over an 8-year period across the three modeled scenarios, by disease stage and overall, and stratified by initial lymph-node status and pathological response after neoadjuvant therapy. The total estimated number of women achieving pCR increased from 4823 in SUS and T-DM1 scenarios (which do not include neoadjuvant pertuzumab) to 8798 in the gold-standard scenario. Across all models, most projected deaths occurred among patients with residual invasive disease.

**Table 2:** Model-projected number of patients and deaths at 8 years, by disease stage and overall, and stratified by initial lymph-node and pCR status, across treatment scenarios for patients with stage II and III HER2-positive breast cancer.

Disease stage	Treatment scenario	Patients, n (95% CI)			Model-projected deaths, n (95% CI)		
		Node negative and pCR	Node positive and pCR	Residual invasive disease	Node negative and pCR	Node positive and pCR	Residual invasive disease
II	SUS	339 (218-483)	809 (533-1127)	4193 (3750–4580)	12 (6-20)	87 (56-126)	759 (620-910)
	T-DM1	339 (218-483)	809 (533-1127)	4193 (3750–4580)	12 (6-20)	87 (56-126)	537 (425-659)
	Gold Standard	618 (456-799)	1477 (1131–1846)	3246 (2742–3726)	22 (13-35)	131 (94-175)	416 (319-524)
III	SUS	1084 (697-1545)	2590 (1706–3606)	13,417 (11,998–14,657)	39 (21-65)	280 (178-402)	2429 (1985–2911)
	T-DM1	1084 (697-1545)	2590 (1706–3606)	13,417 (11,998–14,657)	39 (21-65)	280 (178-402)	1718 (1361–2110)
	Gold Standard	1978 (1460–2557)	4726 (3620–5908)	10,388 (8775–11,922)	71 (41-112)	420 (302-560)	1330 (1021–1678)
Overall	SUS	1423 (915-2028)	3400 (2239–4733)	17,609 (15,748–19,237)	51 (27-86)	367 (234-528)	3188 (2605–3820)
	T-DM1	1423 (915-2028)	3400 (2239–4733)	17,609 (15,748–19,237)	51 (27-86)	367 (234-528)	2254 (1786–2770)
	Gold Standard	2596 (1916–3356)	6202 (4751–7755)	13,634 (11,517–15,648)	93 (53-148)	552 (396-734)	1745 (1340–2202)

CI, confidence interval; pCR: pathological complete response; SUS, Unified Health System; T-DM1: trastuzumab emtansine.

### 3.2 Estimated avoidable deaths

Table 3 details the estimated number and proportion of avoidable deaths across treatment scenarios, overall and stratified by disease stage. In the SUS Scenario, the model-estimated mean number of deaths at 8 years was 3606 (95% CI: 3082–4180). This number was reduced to 2672 (95% CI: 2244–3147) in the T-DM1 scenario and further to 2390 (95% CI: 2034–2790) in the gold-standard scenario. The incorporation of T-DM1 was estimated to avoid 934 deaths (95% CI: 251–1634), representing a 25·89% reduction in mortality compared with the SUS scenario. The gold-standard scenario would avoid 1216 deaths (95% CI: 558–1892), a 33·71% reduction compared with the SUS scenario (Fig. 2). In both scenarios, the reduction rate was consistent between stage II and III disease. Based on the results shown in Table 2, the estimated numbers of avoidable deaths can be analyzed in the subgroups defined by lymph-node status and pathological response. Patients with residual invasive disease following neoadjuvant therapy would have a 29·30% reduction in estimated deaths with T-DM1 incorporation and a 45·26% reduction with the gold-standard regimen, both in comparison with the SUS scenario. In the two subgroups achieving pCR, there would be a higher number of deaths in the gold-standard scenario, but this is due to the increase of the population achieving pCR from the use of neoadjuvant pertuzumab (i.e., the total number of deaths in the gold-standard scenario would still decrease, as demonstrated by the overall results shown in Table 3). When directly comparing the gold-standard and T-DM1 scenarios, the model indicated an additional 283 avoidable deaths (95% CI: 91–510), corresponding to a further 10·59% reduction in mortality, favoring the gold-standard regimen.

**Table 3:** Model-projected deaths and avoidable deaths at 8 years across treatment scenarios in patients with stage II and III HER-positive breast cancer, overall and stratified by disease stage.

Disease stage	N	Model-projected deaths, n (95% CI)			T-DM1 scenario		Gold-standard scenario		Gold-standard vs. T-DM1 scenarios	
		SUS	T-DM1	Gold Standard	Avoidable deaths, n (95% CI)	Avoidable deaths, % (95% CI)	Avoidable deaths, n (95% CI)	Avoidable deaths, % (95% CI)	Avoidable deaths, n (95% CI)	Avoidable deaths, % (95% CI)
II	5341	858 (734-996)	636 (535-750)	569 (485-665)	223 (60-389)	25·96 (6·02-53·00)	290 (133-451)	33·76 (13·35-61·44)	68 (22-122)	10·68 (2·93-22·8)
III	17,091	2748 (2348–3185)	2037 (1709–2398)	1821 (1550–2126)	712 (192-1245)	25·91 (6·03-53·02)	927 (425-1442)	33·73 (13·34-61·41)	215 (70-388)	10·55 (2·92-22·7)
Overall	22,432	3606 (3082–4180)	2672 (2244–3147)	2390 (2034–2790)	934 (251-1634)	25·89 (6·00-53·02)	1216 (558-1892)	33·71 (13·35-61·39)	283 (91-510)	10·59 (2·89-22·73)

CI, confidence interval; SUS, Unified Health System; T-DM1: trastuzumab emtansine.

**Fig. 2:**
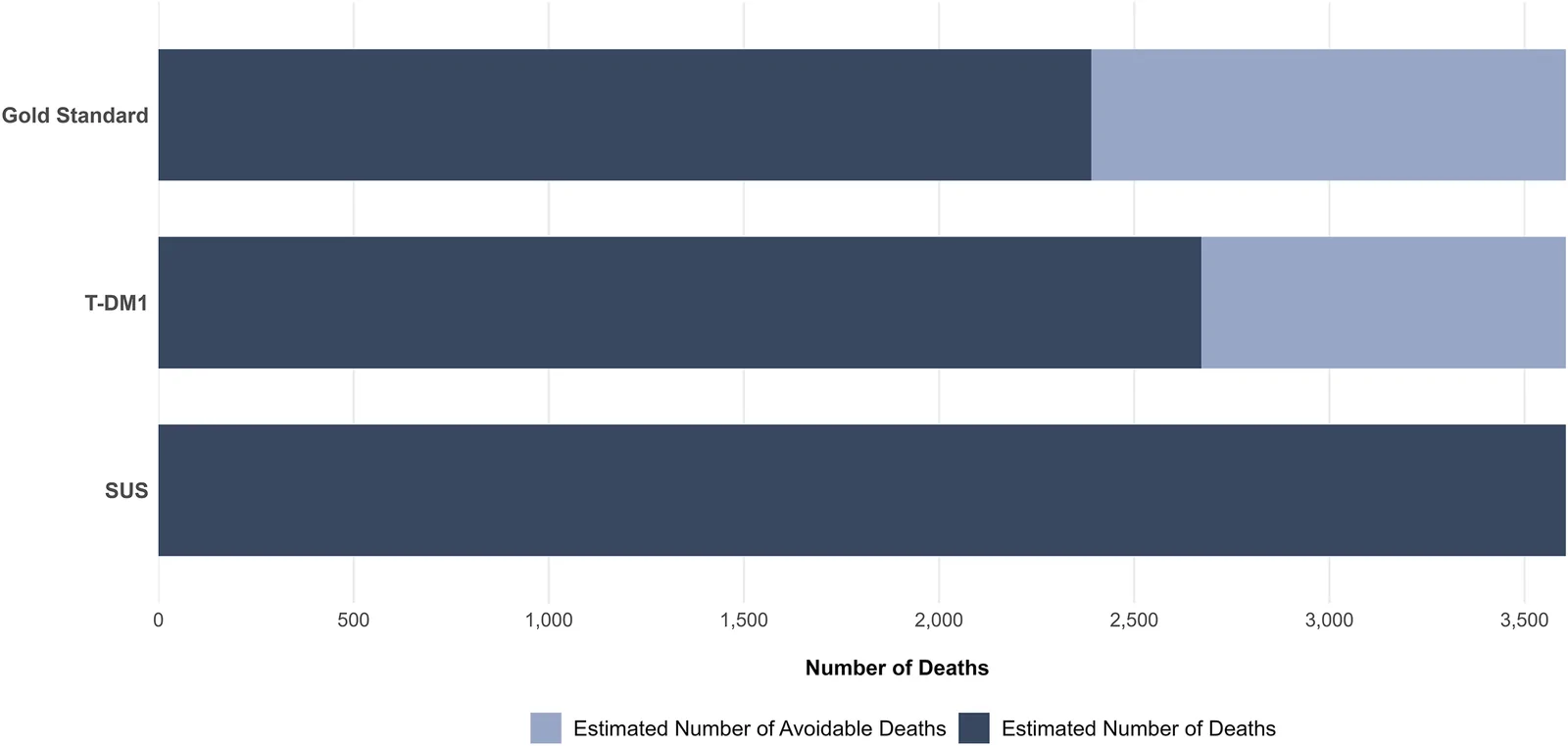
Model-projected number of deaths and avoidable deaths overall at 8 years, in comparison with SUS scenario, according to treatment scenario.

## 4 Discussion

This study suggests that the incorporation of adjuvant T-DM1 with or without perioperative pertuzumab for patients with stage II and III HER2-positive breast cancer could markedly reduce the number of avoidable deaths at 8 years in SUS. We estimated that 934 deaths (a 25·89% reduction) could be prevented through the addition of adjuvant T-DM1 for patients with residual invasive disease after neoadjuvant therapy with trastuzumab, and 1216 deaths (a 33·71% reduction) with the full adoption of the gold-standard regimen, which combines perioperative pertuzumab with trastuzumab and adds adjuvant T-DM1 based on pathological response.

These results align with the well-established survival benefits of T-DM1 and pertuzumab demonstrated in Phase III clinical trials in early-stage disease, including significant improvements in both IDFS and OS [11,12]. Several modeling studies have projected similar benefits with incorporation of those drugs in different settings on a global and regional scale. One epidemiological analysis estimated that, between 2004 and 2033, nearly 768,000 women worldwide with early-stage HER2-positive breast cancer would have avoided metastatic recurrence owing to HER2-targeted therapies — a 26% reduction compared with chemotherapy alone [19]. In the Latin American context, a report from Brazil demonstrated that introducing anti-HER2-targeted therapy for advanced breast cancer could increase the number of women alive after a 2-year follow up: from 808 with chemotherapy alone to 1408 with trastuzumab plus chemotherapy and 1576 with the dual blockade of pertuzumab and trastuzumab plus chemotherapy [25]. In the early-stage setting, the addition of neoadjuvant pertuzumab to a trastuzumab and chemotherapy regimen was estimated to avoid 166 local relapses and 553 distant relapses over 5 years in SUS [18]. Furthermore, an analysis indicated that adjuvant pertuzumab combined with trastuzumab could reduce the development of metastatic disease over 10 and 20 years by 12% and 15% for node-positive disease and by 10% and 15% for hormone receptor negative-disease, respectively [26].

Beyond the clinical impact, these therapies have shown economic feasibility in various healthcare systems [18,[27], [28], [29], [30]]. Cost-effectiveness analyses are particularly relevant for the Brazilian public healthcare system, where health-technology assessment is central to drug incorporation decisions [14]. For instance, a Brazilian study demonstrated that adjuvant T-DM1 is cost-effective versus trastuzumab for treating residual disease after neoadjuvant therapy in both the United States and Brazilian contexts [29]. Similarly, pertuzumab-based dual HER2 blockade has been found to be economically viable, particularly for node-positive and high-risk patients [18,30]. In the neoadjuvant setting, cost-offset analyses projected long-term savings for the Brazilian healthcare system, with a 5-year net reduction of BRL 69·3 million (USD 17·8 million at the time of analysis) [18].

Furthermore, our study highlights a relevant disparity in access to care in Brazil. The model-projected 33·71% reduction in avoidable mortality at 8 years with the gold-standard regimen, in comparison with SUS standard treatment (until November 2025, *i.e.,* prior to delivery to the MoH), confirms the limitations of the public healthcare system. This directly contrasts with the private healthcare sector, which already has regulatory approval and broad access to these HER-2-targeted drugs. Given that approximately 75% of the Brazilian population is covered exclusively by SUS, that 33·71% relative reduction in mortality would imply a substantial impact on public health [15]. It would also be significant in general socioeconomic terms, as women are the main providers for half of the families in Brazil [31]. While our study focuses on systemic treatment, it is well known that other factors also influence the disparity in cancer treatment quality between the two healthcare sectors, such as delayed diagnoses, long travel distances, late treatment initiation limited radiotherapy and surgical capacity, workforce shortages, and limited access to local therapies [3,15]. These health-system capacity constraints were not explicitly incorporated into the model, which assumes that the other components of multidisciplinary cancer care are available and delivered appropriately. Therefore, the model-projected mortality reductions represent the potential benefit of improved systemic treatment under adequate implementation conditions and should not be interpreted as the survival improvement expected from drug incorporation alone. Consequently, some Brazilian analyses have suggested a worse OS for patients with breast cancer treated in the public system compared with those in the private system [32,33].

The proportional reduction in avoidable deaths was similar across stages II and III because of applying the same modelling framework to both groups. However, the absolute benefit was greater in stage III due to its higher prevalence in this analysis, reflecting the typically late cancer diagnosis in Brazil [3]. Given that 5-year OS for stage III HER2-positive breast cancer in Brazil remains only 64·1%, improving access to more effective therapies could have a substantial impact on survival in this high-risk population [4].

The main strengths of this study lie in its nationwide scope and methodological rigor. By using real-world data from DATASUS, which includes all patients treated in the Brazilian public healthcare system, the analysis captures a comprehensive and representative picture of the national population diagnosed with stage II and III HER2-positive breast cancer. The large sample size strengthens the external validity of the findings and allows for robust modeling of outcomes. In addition, the use of simulation and probabilistic sensitivity analyses provided a realistic estimation of uncertainty, enhancing the reliability of the projected mortality reductions. The study also relied on evidence-based clinical parameters derived from relevant Phase II and pivotal Phase III trials, ensuring biological and therapeutic plausibility. Finally, by focusing on avoidable deaths, the analysis offers a clear and policy-relevant perspective on how delayed incorporation of effective therapies impacts survival — an approach that directly supports decision-making within Brazil's health technology assessment framework.

However, this study also has several limitations. First, the model was based on key parameters and OS probabilities derived from relevant and pivotal clinical trials, whose highly selected populations and high-income healthcare settings may not fully reflect SUS patient profiles and outcomes in the Brazilian public healthcare system, where patients often present with more advanced disease and face delays in diagnosis and treatment. Likewise, the model presumed adherence to treatment and completion of the prescribed therapy, which may not occur consistently in real-world settings. As a result, absolute estimates of pCR and long-term survival may be overly optimistic when applied to this context. Second, the model assumes universal use of neoadjuvant systemic therapy for all eligible women with stage II–III HER2-positive breast cancer. Although this reflects guideline-recommended practice, real-world SUS data indicate that a proportion of patients, particularly those with stage II disease, still undergo upfront surgery. Consequently, the model may overestimate both the proportion of patients achieving pCR and the number eligible for escalation with adjuvant T-DM1. Third, despite the use of a large national database (DATASUS), some degree of underreporting or miscoding cannot be excluded, and information on important clinical variables (such as hormone-receptor status, tumor grade, and comorbidities) was not available. In addition, another limitation inherent to analyses based on DATASUS is the absence of direct information on clinical staging and the inability to track patients across healthcare sectors. Although we used a highly specific APAC-AQ code that uniquely identifies patients with HER2-positive, non-metastatic stage II-III breast cancer receiving neoadjuvant therapy within SUS, the database does not allow us to determine whether some patients initiated treatment in the private sector and subsequently migrated to SUS, or conversely began treatment in SUS and continued in the private system. Such transitions, although expected to be infrequent, could lead to incomplete capture of treatment sequences or outcomes and introduce potential misclassification. Fourth, the concept of “avoidable deaths” used in this analysis reflects model-based projections under ideal, guideline-concordant implementation of therapy rather than observed real-world outcomes. These estimates assume timely access, optimal adherence, and effectiveness equivalent to that observed in clinical trials. Therefore, the number of deaths described as “avoidable” should be interpreted as potentially preventable under optimal conditions, not as a prediction of the exact real-world effect following therapy incorporation.

In addition, the model did not explicitly incorporate health-system capacity constraints affecting other components of breast cancer care, including radiotherapy availability, surgical and diagnostic capacity, treatment delays, geographic barriers, and oncology workforce limitations. Furthermore, shorter follow-up OS estimates (e.g., at 5 years) were not used in sensitivity analyses because, at those timepoints, the accrued number of events was insufficient to provide adequate statistical power, given the event-driven design of the pivotal trials and the long natural history of early HER2-positive disease; incorporating such underpowered estimates could introduce artificial volatility into the long-term projections. Matched 8-year OS parameters, based on enough accrued events, therefore provided the most robust basis for the model. Although the same cohort and stage distribution were applied across all treatment scenarios—so that background mortality would be expected to affect them equally—competing causes of death were not modeled separately. Consequently, the results should be interpreted as model-projected all-cause deaths potentially avoidable through improved curative treatment within 8 years, rather than as breast cancer-specific or lifetime mortality estimates. A shorter horizon could miss later treatment benefits, whereas a longer one would require extrapolation and increase uncertainty related to competing mortality.

## 5 Conclusion

In conclusion, incorporating adjuvant T-DM1 and perioperative pertuzumab into the treatment of women with stage II and III HER2-positive breast cancer was projected to substantially reduce potentially avoidable all-cause mortality within 8 years. Beyond the individual clinical benefit, these findings highlight a major opportunity to improve population-level outcomes and address a critical inequity in access to life-saving therapies. Expanding the availability of modern anti-HER2 regimens in SUS would represent an important step toward reducing the survival gap between the public and private sectors. Further evaluations of cost-effectiveness and budgetary impact are warranted to inform evidence-based policy decisions and support timely incorporation of these therapies into national cancer care protocols.

## CRediT authorship contribution statement

**André Mattar:** Writing – review & editing, Writing – original draft, Validation, Supervision, Methodology, Investigation, Funding acquisition, Conceptualization. **Francisco Pimentel Cavalcante:** Writing – review & editing, Writing – original draft, Visualization, Validation, Investigation. **Nelson Francisco Correa-Netto:** Writing – review & editing, Validation, Software, Methodology, Investigation, Formal analysis, Data curation. **Iago Silveira:** Writing – review & editing, Validation, Software, Methodology, Investigation, Formal analysis, Data curation. **Eduardo Baldassin Pavanel:** Writing – review & editing, Validation, Project administration, Methodology, Investigation. **Cintia S. Kurokawa La Scala:** Writing – review & editing, Validation, Methodology, Investigation. **Patricia Klarmann Ziegelmann:** Writing – review & editing, Writing – original draft, Validation, Supervision, Project administration, Methodology, Investigation.

## Ethical approval

This study was based exclusively on secondary analysis of publicly available, de-identified data from the Department of Information and Informatics of the Brazilian Unified Health System (DATASUS). The database contains anonymized administrative health records and does not include any identifiable personal information. According to Brazilian regulations (Resolution No. 510/2016 of the National Health Council), research using publicly accessible, de-identified data does not require review or approval by a Research Ethics Committee. Therefore, formal ethical approval and informed consent were waived.

## Funding

Produtos 10.13039/100004337Roche Químicos e Farmacêuticos, Brazil.

## Authors' disclosures of potential conflicts of interest

**AM** declares consulting fees from Roche and Eli Lilly; payment or honoraria for lectures, presentations, speakers’ bureaus, manuscript writing or educational events from Merck, Roche, Eli Lilly and AstraZeneca; and received support for attending meetings and/or travel from Roche.

**FPC** declares consulting fees from AstraZeneca and Roche; payment or honoraria for lectures, presentations and speakers’ bureaus from Merck, Roche, AstraZeneca, Daiichi Sankyo, and Eli Lilly; manuscript writing from Roche; and received support for attending meetings and/or travel from Roche, Merck, and AstraZeneca.

**NFC-N** declares employment: Produtos Roche Quimicos e Farmaceuticos, Brazil.

**IS** declares employment: Produtos Roche Quimicos e Farmaceuticos, Brazil.

**EBP** declares employment: Produtos Roche Quimicos e Farmaceuticos, Brazil.

**CKS** declares employment: Produtos Roche Quimicos e Farmaceuticos, Brazil.

**PKZ** declares consulting fees from Roche.
