# Supplementary material for: Impact of anti-HER2 therapies on potentially avoidable deaths from stage II and III HER2-positive breast cancer in the Brazilian public healthcare system
Source: Breast. 2026 Oct 1;90:104940. doi: 10.1016/j.breast.2026.104940 (PMC13661201; doi:10.1016/j.breast.2026.104940)
Supplement: Supplementary file 1 [file mmc1.docx]

**Supplementary Material to “Impact of Anti-HER2 Therapies on Potentially Avoidable Deaths from Stage II and III HER2-Positive Breast Cancer in the Brazilian Public Healthcare System”**

**Supplementary Material S1. High-cost chemotherapy procedure authorizations (APAC-AQ) codes for study eligibility**

Codes and description of eligible outpatient chemotherapy regimens recorded in APAC-AQ files of Ambulatory Information System (SIA-SUS**)**:

- 03.04.05.027-0 – Polychemotherapy for HER2-positive breast carcinoma, stage II (adjuvant)
- 03.04.05.030-0 – Monochemotherapy for HER2-positive breast carcinoma, stage II (adjuvant)
- 03.04.04.018-5 – Polychemotherapy for HER2-positive breast carcinoma, stage III (neoadjuvant)
- 03.04.05.028-8 – Polychemotherapy for HER2-positive breast carcinoma, stage III (adjuvant)
- 03.04.05.031-8 – Monochemotherapy for HER2-positive breast carcinoma, stage III (adjuvant)

The two procedures listed below were retained because, during the exclusion step in which patients with a non-HER2-related initial therapeutic procedure were removed (Supplementary Figure 1), these specific regimens were not classified as exclusionary. Patients who initiated treatment with one of these procedures and subsequently received HER2-targeted therapy remained eligible and were thus preserved in the analytical cohort.

- 03.04.04.002-9 – Polychemotherapy for breast carcinoma (neoadjuvant)
- 03.04.05.007-05 – Monochemotherapy for breast carcinoma, stage II (adjuvant)

**Supplementary Material S2. Model parameters for projections of potentially avoidable deaths**

**Supplementary Table 1.** Model parameters for projections of potentially avoidable deaths

| **Parameter** | **Measure of central tendency** | **CI** | | **Parameters of beta distribution** | **Observations** | **Reference** |
| --- | --- | --- | --- | --- | --- | --- |
|  |  | **Lower limit** | **Upper limit** |  |  |  |
| Proportion of lymph node-negative disease | 0.295 | 0.251 | 0.339 | α = 123  β = 294 | Measure of central tendency and CI calculated based on data presented in the reference publication, considering the entire study sample. | NeoSphere  (Gianni et al., 2012) |
| Proportion of pCR – Neoadjuvant treatment with trastuzumab | 0.215 | 0.141 | 0.305 | α = 23  β = 84 | Measure of central tendency and CI obtained from the reference publication. | NeoSphere  (Gianni et al., 2012) |
| Proportion of pCR – Neoadjuvant treatment adding pertuzumab | 0.393 | 0.300 | 0.492 | α = 42  β = 65 | Measure of central tendency and CI obtained from the reference publication. | NeoSphere  (Gianni et al., 2012) |
| 8-year OS – Adjuvant treatment with trastuzumab for lymph node-positive and pCR | 0.892 | 0.873 | 0.908 | α = 1077.0  β = 130.4 | Measure of central tendency and CI estimated based on data presented in the reference publication. | APHINITY  (Loibl et al., 2024) |
| 8-year OS – Adjuvant treatment adding pertuzumab for lymph node-positive and pCR | 0.911 | 0.893 | 0.926 | α = 1041.3  β = 101.7 | Measure of central tendency and CI estimated based on data presented in the reference publication. | APHINITY  (Loibl et al., 2024) |
| 8-year OS – Adjuvant treatment with trastuzumab for lymph node-negative and pCR | 0.964 | 0.946 | 0.976 | α = 570.2  β = 21.3 | Measure of central tendency and CI estimated based on data presented in the reference publication. | APHINITY  (Loibl et al., 2024) |
| 8-year OS – Adjuvant treatment with trastuzumab for invasive residual disease | 0.819 | 0.790 | 0.849 | α = 535.1  β = 118.3 | Measure of central tendency and CI calculated based on anonymized study data, provided by the sponsor study. | KATHERINE  (Geyer et al., 2025) |
| 8-year OS – Adjuvant treatment with T-DM1 for invasive residual disease | 0.872 | 0.847 | 0.897 | α = 597.3  β = 87.7 | Measure of central tendency and CI calculated based on anonymized study data, provided by the study sponsor. | KATHERINE  (Geyer et al., 2025) |

CI, confidence interval; OS, overall survival; pCR, pathological complete response; T-DM1, trastuzumab emtansine.

**Supplementary Material S3. Model structure, notation and equations**

**S3.1 Notation**

**Supplementary Table 2.** Symbols and definitions used in equations

| **Symbol** | **Definition** |
| --- | --- |
| *j* | Disease stage at diagnosis, *j* ∈ {II, III} |
| *s* | Treatment scenario, *s* ∈ {SUS, T-DM1, Gold standard} |
| *g* | Model branch, *g* ∈ {N-/pCR, N+/pCR, non pCR} |
| *m* | Simulation iteration, *m* = 1, ..., *M* with *M* = 100,000 |
| *N_j_* | Number of eligible patients with stage j disease identified in DATASUS (fixed, non-stochastic) |
| $p_{N-}^{(m)}$ | Probability of node-negative disease at diagnosis, iteration *m* |
| $q_{s}^{(m)}$ | pCR rate under the neoadjuvant regimen of scenario *s*, iteration *m* |
| $p_{g,s}^{(m)}$ | Probability of belonging to branch *g* under scenario *s*, iteration *m* |
| $S_{g,s}^{(m)}$ | Branch- and scenario-specific 8-year overall survival probability, iteration *m* |
| $D_{g,s}^{(m)}$ | Projected deaths within 8 years in branch *g*, stage *j*, scenario *s* |
| $D_{s}^{(m)}$ | Total projected deaths within 8 years under scenario *s* |
| $A_{a,b}^{(m)}$ | Potentially avoidable deaths, alternative scenario *a* versus reference scenario *b* |
| $R_{a,b}^{(m)}$ | Proportional reduction in deaths, scenario *a* versus scenario *b* |
| $\hat{D}_{a,b}$, $\hat{A}_{a,b}$ , $\hat{R}_{a,b}$ | Point estimates (means across all *M* iterations) |

**S3.2 Branch membership probabilities**

Node status at diagnosis is independent of the neoadjuvant regimen, whereas the pCR rate is scenario-specific (trastuzumab-based regimens in the SUS and T-DM1 scenarios; trastuzumab plus pertuzumab in the gold-standard scenario). For each iteration *m*:

$$p_{N-/pCR, s}^{(m)}= p_{N-}^{(m)} \times q_{(s)}^{(m)}$$

$$p_{N+/pCR, s}^{(m)}= \left( {1- p}_{N-}^{(m)} \right) \times q_{(s)}^{(m)}$$

$$p_{non pCR, s}^{(m)}= 1-q_{(s)}^{(m)}$$

with, by construction, $\sum_{g} p_{g,s}^{(m)}=1$ for every scenario *s* and iteration *m*.

The expected number of deaths within the 8-year horizon in each branch is the product of the number of patients allocated to that branch and the branch-specific 8-year all-cause mortality probability, $1- S_{g,s}^{(m)}$:

$$D_{g,j,s}^{(m)}= N_{j} \times p_{g,s}^{(m)} \times\left( 1- S_{g,s}^{(m)} \right)$$

Total deaths per scenario are obtained by summing across branches and stages:

$$D_{s}^{(m)}= \sum_{j} \sum_{g} D_{g,j,s}^{(m)}$$

**S3.4 Potentially avoidable deaths and proportional reduction**

For an alternative scenario a compared with a reference scenario b, both the potentially avoidable deaths and the proportional reduction were computed within each iteration *m*, so that parameter uncertainty is propagated consistently to all outputs:

$$A_{a,b}^{(m)}= D_{(b)}^{(m)}- D_{a}^{(m)}$$

$$R_{a,b}^{(m)}= \frac{A_{a,b}^{(m)}}{D_{b}^{(m)}}= \frac{D_{b}^{(m)}- D_{a}^{(m)}}{D_{b}^{(m)}}$$

Point estimates were obtained as the mean across the *M* = 100,000 iterations:

$$\hat{D}_{a,b}= \frac{1}{M}\sum_{m=1}^{M} D_{a,b}^{(m)}$$

$$\hat{A}_{a,b}= \frac{1}{M}\sum_{m=1}^{M} A_{a,b}^{(m)}$$

$$\hat{R}_{a,b}= \frac{1}{M}\sum_{m=1}^{M} R_{a,b}^{(m)}$$

The reference scenario b (i.e., the scenario with fewer treatment options and higher projected mortality) is always placed in the denominator, so that a positive $R_{a,b}^{(m)}$, denotes a reduction in deaths achieved by adopting scenario a instead of b. Three comparisons were evaluated:

Supplementary Table 3. Reference scenario used in the denominator of the proportional-reduction calculation for each pairwise comparison.

| **Comparison (a vs b)** | **Reference in Denominator (D*_b_*)** |
| --- | --- |
| T-DM1 vs SUS | T-DM1 vs SUS |
| SUS | SUS |
| Gold standard vs SUS | Gold standard vs SUS |

**S3.5 Probabilistic sensitivity analysis algorithm**

1. For each interaction *m* = 1, ..., 100,000:
2. Draw $p_{N-}^{(m)} \sim Beta\left( \alpha,\beta\right)$.
3. Draw the scenario-specific pCR rates $q_{s}^{(m)} \sim Beta\left( \alpha,\beta\right)$.
4. Draw the branch and scenario-specific 8-year OS probabilities $S_{g,s}^{(m)} \sim Beta\left( \alpha,\beta\right)$
5. Compute branch membership probabilities (S3.2).
6. Compute branch-level and total deaths for each scenario (S3.3).
7. Compute avoidable deaths and the proportional reduction for each pairwise comparison (S3.4).

**Supplementary Material S4. Supplementary Figures**

**Supplementary Figure 1.** Numbers of patients registered in the database and analyzed, with reasons for exclusion.

APAC-AQ: high-cost chemotherapy procedure authorizations; HER2: human epidermal growth factor receptor 2; ICD-10: international classification of diseases-10; SIA-SUS: ambulatory information system.

**Supplementary Figure 2.** Patient distribution according to disease stage and period of treatment initiation.
